# Varying Forms of Childhood Sexual Abuse and Late-Life Depression Among Older Adults Living with HIV

**DOI:** 10.1007/s10461-025-04920-7

**Published:** 2025-10-10

**Authors:** Monique J. Brown, Medinat Omobola Osinubi, Amandeep Kaur, Prince Nii Ossah Addo, Daniel Amoatika, David Owiredu, Elizabeth Crouch, Steven A. Cohen

**Affiliations:** 1 Department of Epidemiology and Biostatistics, Arnold School of Public Health, University of South Carolina, 915 Greene Street Discovery I, 435C, Columbia, SC 29208 USA; 2 South Carolina SmartState Center for Healthcare Quality, Arnold School of Public Health, University of South Carolina, Columbia, SC USA; 3 Rural Health Research Center, Arnold School of Public Health, University of South Carolina, Columbia, SC USA; 4 Office for the Study on Aging, Arnold School of Public Health, University of South Carolina, Columbia, SC USA; 5 Centre for Health Systems Research & Development, University of the Free State, Bloemfontein, South Africa; 6 Health Services Policy and Management, Arnold School of Public Health, University of South Carolina, Columbia, SC USA; 7 Department of Public Health, University of Rhode Island, Kingston, Rhode Island

**Keywords:** CSA, Depressive symptoms, Older adults

## Abstract

Childhood sexual abuse (CSA) has been linked to depression in adulthood. However, studies examining the potential link between CSA and depression in older adulthood are limited. Therefore, the aim of this study was to examine the association between CSA and late-life depression among older adults living with HIV (OALH) using two measures of depression. Data were obtained from OALH attending an immunology clinic in South Carolina (*n* = 91). Multivariable logistic and multiple linear regression models were used to determine the associations between varying forms of CSA and late-life depression. All forms of CSA were associated with self-report depression (for e.g., adjusted OR for being touched intimately: 5.46; 95% CI: 1.66-18.0) while only overall CSA (adjusted B: 3.32; 95% CI: 0.36, 6.29 and experiencing genital rubbing (adjusted B for experiencing genital rubbing: 3.88; 95% CI: 1.02, 6.74) were associated with Patient Health Questionnaire-9 depressive symptoms. Trauma-informed interventions addressing specific types of CSA may help to reduce depression among OALH.

## Introduction

Childhood sexual abuse (CSA) is a prevalent event with population-based prevalence estimates ranging from 6 to 32% [1]. CSA has emerged as a risk factor for various adverse health and mental health sequelae in adults. For instance, CSA has been associated with mental health problems such as depression, anxiety, and posttraumatic stress disorder (PTSD), as well as substance use disorders [2–4]. About 30 to 53% of people living with HIV (PLWH) have experienced sexual violence during childhood and adolescence [4, 5].

For PLWH, traumatic experiences such as CSA can also influence the development and continuation of anxiety and depressive symptoms [4]. Published data showed that heterosexual women living with HIV and CSA who reported higher HIV-related shame and higher sexual abuse-related shame were more likely to report more anxiety and depressive symptoms [4]. However, heterosexual women living with HIV and CSA who reported higher posttraumatic growth were more likely to report fewer anxiety and depressive symptoms [4]. Findings of a study by James et al. (2022) showed that PLWH who have experienced CSA have stated that they desired intimate relationships but experienced rejection while trying to establish intimate relationships, which negatively impacted their health and quality of life [6]. In this study, participants expressed feelings of helplessness or vulnerability due to loneliness or being alone, inability to leave an intimate partner despite being disrespected and taken advantage of, and difficulty dealing with HIV status, all of which negatively impacted their health and well-being [6].

CSA tends to have more severe consequences compared to other adverse childhood experiences (ACEs). CSA was the primary driver of the association between ACEs and depressive symptoms among pregnant women living with HIV in Malawi [7]. Sexual abuse during childhood had the largest effect and was statistically significantly associated with all depressive symptoms except for somatic complaints [7]. Bullying and emotional neglect were also associated with depressive symptoms, but to a lesser extent compared to CSA [7].

CSA has long-lasting impacts and might continue to affect older adults. Talbot and colleagues found that CSA was associated with more incredible pain, lower physical functioning, and adverse health outcomes among psychiatric patients aged 50 and older [8]. CSA has also been linked to poor physical and mental health among adults aged 60 and older [9]. CSA might result in abstaining from sexual activities [6] and impact the overall quality of life in older adulthood [9].

The prevalence of older adults living with HIV (OALH) continues to increase due to the improvements in antiretroviral therapy (ART) and new cases among this population. Approximately 41% of people with HIV are aged 55 and older [10], and more than half (54%) are aged 50 and older [11]. The HIV rates among people aged 55 and older have remained stable in 2022 compared to 2018 [11]. OALH may face many challenges including a higher prevalence of comorbidities, including cardiovascular disease, cancer and diabetes [12]. Additional challenges that OALH may face include late diagnosis during the course of the disease [13], polypharmacy [14], “double stigma” [15], social isolation [15] and depression [14].

Previous research has identified protective and risk factors for depression among OALH. Social support has been found to be associated with fewer depressive symptoms among OALH. However, greater HIV-related stigma, discrimination in medical settings and structural barriers have been shown to be associated with greater depressive symptoms, specifically among OALH. Depression has been identified as a potential barrier to successful cognitive aging [16], and neurological and functional impairment [17] among OALH.

Nevertheless, one group that is understudied with respect to childhood sexual trauma are OALH [18]. Brown et al. reported a 32% prevalence of CSA among OALH [5]. This study found no statistically significant differences in the CSA history by any sociodemographic characteristic [5]. However, participants who reported CSA had statistically significantly higher mean levels of depression than those who did not [5].

Childhood sexual abuse (CSA) is a global problem with serious repercussions for survivors in various domains of adult interpersonal functioning [19]. CSA has been previously linked to depression in adulthood [7, 9]. Yet, studies examining CSA and depression in late-life are limited, especially among older adults living with HIV (OALH). In addition, studies examining different forms of CSA and depression are scarce. Therefore, the aims of this study were two-fold: (1) To examine the association between CSA and depression among OALH; and (2) To assess the relationship between varying forms of CSA and depression among OALH.

## Methods

### Data Source and Study Population

Data were obtained from 91 adults aged 50 and older living with HIV and attending an immunology clinic in South Carolina, and were collected between April and June 2021. Clinic staff assisted research assistants in recruiting potential participants for the current study based on age eligibility. Informed consent was obtained from individuals who expressed an interest in the study and were eligible (aged 50 and older and living with HIV). Participants were compensated with a $40 gift card [5]. The study protocol was approved by the University of South Carolina Institutional Review Board.

### Primary Measures

*Childhood Sexual Abuse (CSA). CSA* was operationalized using six questions from the Early Trauma Inventory-Self Report Form [20]. These questions asked about experiences with CSA before aged 18 years old: (1) being touched in an intimate/private part of the body; (2) experiencing someone rubbing their genitals against the participant; (3) forced/coerced to touch another person in an intimate/private part of their body; (4) having genital sex against their will; (5) performing oral sex on someone against the participant’s will; and (6) forced/coerced to kiss someone in an affectionate way. Responses were yes vs. no. We operationalized CSA as reporting any of these experiences vs. not reporting any CSA history. We also examined each form of CSA separately.

*Depression* was measured in two ways: (1) Self-reported answer to the question: “Do you have depression?”; and (2) Using the Patient Health Questionnaire-9 (PHQ-9) [21, 22]. The question on depression was taken from the Self-Administered Comorbidity Questionnaire (SCQ) [23], which asks about health challenges of participants. Examples of items asked in the PHQ-9 were: “feeling tired or having little energy”, and “poor appetite or overeating”. Responses were obtained on a four-point Likert-type scale with answers ranging from 0 to 3 (“Not at all” to “Nearly every day”). Answers from the PHQ-9 obtained data on depressive symptoms in the past two weeks. Scores were summed to receive a total depression score. The one-item measure on depression was asked to determine if self-reported depression would align with the reports from the PHQ-9 and if the way depression is measured would change the study results.

*Sociodemographic confounders* considered were age, gender, race and educational attainment. We examined sociodemographic variables that have been linked to CSA and depression but are not considered to be in the mediational pathway between CSA and depression. For example, age, gender, race, and educational attainment differences have been found with respect to CSA status where younger adults, women, and individuals attaining some college education or lower tended to report a CSA history compared to older adults, men and individuals with at least a college degree [24]. Previous studies have found racial differences in having a CSA history to be mixed with statistically significant differences (Black and Latino/Hispanic individuals having a higher rate of CSA histories) [25] and a lack thereof [24]. In addition, sociodemographic differences have been found in depression where younger, women, individuals with lower educational attainment and Black and White individuals tend to report depression compared to older individuals, men, individuals with higher educational attainment and Other and Hispanic individuals [26].

### Analytic Approach

Crude and adjusted multivariable logistic regression models were used to determine the association between varying forms of CSA and self-reported depression during older adulthood. Simple and multiple linear regression models were used to determine the association between varying forms of CSA and PHQ-9 depressive symptoms during older adulthood. Adjusted models (both logistic and linear) controlled for sociodemographic characteristics (age, gender, race, and education). Statistical significance was set at *p* < 0.05. All data were analyzed using SAS version 9.4 (SAS Institute, Cary, North Carolina).

## Results

Table 1 shows the distribution of sociodemographic characteristics, CSA status and self-reported depression among OALH. Approximately 60% were male, almost 83% were between the ages of 50 and 64 years, and about 70% identified as Black. The mean (SD) age of the sample was 58.1 (6.7) years. Approximately one in three reported being CSA survivors (32%) and close to four in ten reported depression (37%). Almost 47% had a high school education or lower educational attainment and close to one-third reported being employed. Approximately 10% reported earning US$500 or less per month and the majority (62%) had been living with HIV for more than 20 years. Close to 40% self-reported depression. Three in four responded to have at least moderate/moderately severe/severe depression based on the PHQ-9 (data not shown). CSA survivors tended to be younger based on the mean age (55.2 vs. 59.4 years). Using the Fisher’s exact *p*-value, OALH who reported a CSA history also tended to be self-report depression. There were statistically significant differences in self-reported depression by mean age, race, employment and CSA status where participants reporting depression tended to be younger (56.1 vs. 59.5 years), White, unemployed and reported a CSA history.

Table 2 shows the relationship between varying forms of CSA and self-reported late-life depression from the logistic regression models. All forms of CSA were associated with higher odds of late-life depression. For example, individuals who reported being touched intimately were approximately 5.5 times more likely to report late-life depression compared to OALH who did not experience CSA (adjusted OR: 5.46; 95% CI: 1.66–18.0.66.0). OALH who reported experiencing forced genital sex against their will were almost 4 times more likely to report late life depression compared to OALH who did not experience CSA (adjusted OR: 3.77; 95% CI: 1.11–12.8). OALH who were forced or coerced to kiss someone had 22 times higher odds of reporting late-life depression compared to OALH who did not report CSA (adjusted OR: 22.7; 95% CI: 2.51–205.3).

Table 3 shows the relationship between varying forms of CSA and depressive symptoms using the PHQ-9 from the linear regression models. All forms of CSA were associated with higher odds of late-life depression, though not all forms achieved statistical significance. In the adjusted models, overall CSA was associated with higher likelihood of late-life depression (adjusted B: 3.32; 95% CI: 0.36, 6.29). For example, individuals who experienced any form of CSA, on average, had a three-point increase in depression score on the PHQ-9. Experiencing genital rubbing was also associated with a higher likelihood of late-life depression (adjusted B: 3.88; 95% CI: 1.02, 6.74). Therefore, individuals who reported this form of CSA had, on average, a four-point increase in depressive symptoms as measured by the PHQ-9.

**Table 1:** Distribution of sociodemographic characteristics, childhood sexual abuse (CSA) and self-reported depression among older adults living with HIV

Characteristic	Total *N* (%)	CSA *N* (%)	No CSA *N* (%)	Fisher’s Exact *P*-value	Depression *N* (%)	No Depression *N* (%)	Fisher’s Exact *P*-value
Gender				1.00			0.452
Male	50 (58.8)	15 (57.7)	32 (58.2)		18 (60.0)	30	
Female	33 (38.8)	10 (38.5)	22 (40.0)		11 (36.7)	21	
Transgender	2 (2.4)	1 (3.9)	1 (1.8)		1 (3.3)	0 (0.0)	
Age (Mean, SD)	58.1, 6.7	55.2, 4.2	59.4, 7.4	0.002*	56.1, 6.1	59.5, 6.8	0.026*
50–64	66 (82.5)	23 (92.0)	39 (76.5)	0.125	26 (92.9)	36 (75.0)	0.068
65+	14 (17.5)	2 (8.0)	12 (23.5)		2 (7.1)	12 (25.0)	
Race				0.187			**0.048**
Black	59 (68.6)	15 (27.7)	40 (71.4)		16 (53.3)	38 (74.5)	
White	26 (30.2)	10 (38.5)	16 (28.6)		14 (46.7)	12 (23.5)	
Other	1 (1.2)	1 (3.9)	0 (0.0)		0 (0.0)	1 (2.0)	
Education				0.446			0.765
< High School	17 (19.8)	9 (34.6)	27 (50.0)		5 (16.7)	11 (21.6)	
High School	23 (26.7)	11 (42.3)	14 (25.9)		9 (30.0)	13 (25.5)	
Some College	26 (30.2)	3 (11.5)	8 (14.8)		10 (33.3)	13 (25.5)	
Bachelors/Post-Grad	20 (23.3)	3 (11.5	5 (9.3)		6 (20.0)	14 (27.5)	
Employed				0.798			**0.012**
Yes	28 (32.6)	7 (26.9)	18 (32.1)		4 (13.3)	21 (41.2)	
No	58 (67.4)	19 (73.1)	38 (67.9)		26 (86.7)	30 (58.8)	
Income				0.505			0.857
≤$500	8 (9.5)	2 (8.3)	5 (8.9)		3 (10.7)	4 (7.8)	
$501 to $1,000	27 (32.1)	9 (37.5)	18 (32.1)		9 (32.1)	18 (35.3)	
$1001 to $2,000	32 (38.1)	6 (25.0)	23 (41.1)		11 (39.3)	17	
$2,000+	17 (20.2)	7 (29.2)	10 (17.9)		5 (17.9)	12	
Years Since Diagnosis				0.317			0.826
≤5 years	4 (5.4)	1 (4.0)	3 (6.4)		1 (3.7)	2 (4.4)	
>5 years to ≤10 years	5 (6.8)	3 (12.0)	2 (4.3)		2 (7.4)	3 (6.7)	
>10 years to ≤20 years	19 (25.7)	4 (16.0)	15 (31.9)		5 (18.5)	13 (28.9)	
>20 years	46 (62.2)	17 (68.0)	27 (57.5)		19 (70.4)	27 (60.0)	
Self-Reported Depression				**<0.001**	–	–	–
Yes							
No	30 (37.0)	18 (69.2)	12 (23.1)				
	51 (63.0)	8 (30.8)	40 (76.9)				
CSA		–	–	–			**<0.001**
Yes	26 (31.7)				18 (60.0)	8 (16.7)	
No	56 (68.3)				12 (40.0)	40 (83.3)	

Bolded *p* values are statistically significant at *p* < 0.05

*Welch’s p-value

**Table 2:** Association between childhood sexual abuse and self-reported late-life depression among older adults living with HIV

	Model 1		Model 2	
Types of CSA	OR	95% CI	Adjusted OR	95% CI
Overall CSA	**7.50**	**2.62–21.5**	**6.30**	**1.90–20.9**
Being touched intimately	**6.00**	**2.12–17.0**	**5.46**	**1.66–18.0**
Experiencing genital rubbing	**8.57**	**2.82–26.1**	**8.07**	**2.37–27.5**
Forced or coerced to touch another person intimately	**4.81**	**1.64–14.1**	**4.04**	**1.24–13.2**
Genital sex against will	**5.25**	**1.79–15.4**	**3.77**	**1.11–12.8**
Forced or coerced to perform oral sex	**14.2**	**2.87–70.0**	**13.0**	**2.37–71.0**
Forced or coerced to kiss someone	**25.0**	**3.00–208.3**	**22.7**	**2.51–205.3**

Bolded OR and 95% confidence intervals are statistically significant at *p* < 0.05

Model 1: Crude logistic regression model

Model 2: Adjusted logistic regression model controlling for sociodemographic confounders

**Table 3:** Association between childhood sexual abuse and depressive symptoms (Patient health Questionnaire-9) among older adults living with HIV

	Model 1		Model 2	
Types of CSA	B	95% CI	Adjusted B	95% CI
Overall CSA	**3.48**	**0.70**,** 6.26**	**3.32**	**0.36**,** 6.29**
Being touched intimately	2.73	− 0.13, 5.59	2.48	− 0.53, 5.49
Experiencing genital rubbing	**4.11**	**1.28**,** 6.95**	**3.88**	**1.02**,** 6.74**
Forced or coerced to touch another person intimately	**3.59**	**0.62**,** 6.56**	2.78	− 0.31, 5.87
Genital sex against will	2.05	− 0.96, 5.06	1.37	−1.88, 4.62
Forced or coerced to perform oral sex	3.44	− 0.10, 6.97	2.61	− 0.97, 6.19
Forced or coerced to kiss someone	3.22	− 0.59, 7.03	2.75	− 1.06, 6.57

Bolded B and 95% confidence intervals are statistically significant at *p* < 0.05

Model 1: Crude linear regression model

Model 2: Adjusted linear regression model controlling for sociodemographic confounders

## Discussion

To our knowledge, no study has examined the associations between varying forms of CSA and late-life depression among OALH. The forms of CSA addressed in the current study included: being touched in an intimate/private part of the body; experiencing someone rubbing their genitals against the participant; forced/coerced to touch another person in an intimate/private part of their body; having genital sex against their will; performing oral sex on someone against the participant’s will; and forced/coerced to kiss someone. The main findings were that all forms of CSA were associated with self-reported late-life depression. However, associations between CSA forms and depressive symptoms based on the PHQ-9 varied depending on the CSA variable.

Previous studies have also shown a relationship between CSA and depression among varying populations, including a nationally representative sample of US adults [2, 3], among Chinese youth [27], and among Canadian men [28]. For example, Brown et al., found that depression mediated the relationship between CSA and sexual health outcomes and behaviors in adulthood [2, 3]. Li et al., 2023 found that factors such as sad mood were central to experiencing depression among CSA survivors [27]. After adjusting for confounders, Moorman & Romano (2023) found that CSA survivors had lower psychological functioning with a higher likelihood of depression [28].

In the current study, it is also interesting that all forms of CSA were associated with self-report of depression while only overall CSA and experiencing genital rubbing were associated with the PHQ-9 report of depressive symptoms. Indeed, previous studies have shown significant disagreement between self-report questionnaires (such as the PHQ-9) and patient-rated views of mood changes [29]. *Post hoc* analyses showed that all participants who responded affirmatively to the one-item depression measure had at least moderate depressive symptoms on the PHQ-9. These findings suggest that it is crucial to determine an individual’s subjective report of mental health outcomes as these results may differ based on questionnaires or an objective perspective of their mental health.

It is important to note the impact that CSA may have on OALH across the life course. Our findings suggest that CSA may have lingering effects for this population and may be linked to depression even in later life. While studies have linked CSA to depression in younger individuals [30, 31], negative self-perceptions of CSA survivors can result in depression among older adults [32]. An inaccurate diagnosis of a history of sexual abuse as dementia or cognitive impairment can act as a significant barrier to mental health treatment and assistance to older CSA survivors [32]. Addressing CSA is also crucial as unresolved trauma survivors may also be at risk of revictimization [32].

Our findings on the association between CSA and late-life depression align with previous research though in different populations [33, 34]. Easton et al., found that men who had a CSA history had greater depressive symptoms compared to men without a history of CSA. In addition, the authors also found that depressive symptoms decreased over time for both CSA survivors and those with no history; and social support moderated the effect between CSA and depressive symptoms [34]. Cook et al., reported that while older sexual and gender minority men reported lower rates of adult sexual assault compared to younger men, the groups did not differ on the experience of CSA or mental health treatment [33]. Cook and colleagues found that there was a need to address mental health challenges related to histories of sexual assault among middle-aged and older individuals using age-sensitive outreach and education [33]. This outreach to older CSA survivors should also consider the myriad physical and psychosocial factors (such as comorbidities and social isolation) that OALH face, which may also contribute to depressive symptoms and impact the overall quality of life among this population.

There are some limitations to be considered in interpreting the results of the current study. First, all individuals were receiving HIV care at an immunology clinic, so results might not be reflective of individuals not in HIV care. Second, the sample size was relatively small, and larger studies are needed to determine if the findings here persist. Third, the participants in the current study were relatively younger when considering the range of ages of older adults. The mean age was 58 years and the majority of participants were under 65 years of age. Fourth, we were unable to determine the frequency of the CSA event(s), and this may have a crucial role to play in the association with late-life depression. Fifth, we also did not have data on the age of CSA exposure, which can also play an important role in associated outcomes across the life course.

## Conclusion

The study showed that varying forms of CSA were associated with self-report of depression while the relationship between these forms of CSA varied with PHQ-9 depressive symptoms based on the CSA form assessed. It is important not only to consider self-report questionnaires, but also patient-rated views of depressive symptoms. Future research should use larger sample sizes of OALH and to determine if these associations persist for OALH. Trauma-informed intervention programs addressing CSA may decrease late-life depression and/or depressive symptoms among OALH.

## Data Availability

Data can be obtained by e-mailing Dr. Monique J. Brown (brownm68@mailbox.sc.edu).

## Code Availability

Codes can be obtained by e-mailing Dr. Monique J. Brown (brownm68@mailbox.sc.edu).
